# Assessment of adipokines relationships with cardiovascular risk markers in relation to body indices in normoglycemic males

**DOI:** 10.12669/pjms.291.2913

**Published:** 2013

**Authors:** Syed Shahid Habib, Khlalid A Al Regaeiy, Laila Al Dokhi

**Affiliations:** 1 Syed Shahid Habib, FCPS, Department of Physiology, College of Medicine, King Saud University, Riyadh-11461, Kingdom of Saudi Arabia.; 2 Khlalid A Al Regaeiy, PhD, Department of Physiology, College of Medicine, King Saud University, Riyadh-11461, Kingdom of Saudi Arabia.; 3 Laila Al Dokhi, PhD, Department of Physiology, College of Medicine, King Saud University, Riyadh-11461, Kingdom of Saudi Arabia.

**Keywords:** Body composition, Fat mass, Body fat percentage, High sensitivity C reactive protein, Lipoprotein(a)

## Abstract

***Objectives:*** To evaluate the phenotypic relationship between obesity indices, resistin, adiponectin and cardiovascular risk markers in normoglycemic healthy individuals.

***Methodology:*** This cross-sectional study was conducted in the Department of Physiology College of Medicine, King Saud University, Riyadh. A total of 120 male subjects were selected for the study. All subjects underwent analysis of body composition, glucose, glycosylated hemoglobin (HbA1c), lipids, adiponectin, resistin, lipoprotein(a) and high sensitivity C reactive protein (hsCRP).

***Results:*** Body mass index (BMI) (r=0.326, p < 0.001), body fat mass (BFM) (r=0.377, p < 0.001), body fat percentage (BF%) (r=0.326, p < 0.001), waist hip ratio (WHR) (r=0.402, p < 0.001) and basal Insulin levels (r=0.217, p=0.018) were positively correlated with hsCRP. However, serum adiponectin levels (r=0.189, p=0.0391) were negatively correlated with hsCRP. Adiponectin levels were significantly lower in obese compared to non obese subjects (p=0.0551). Keeping hsCRP as dependant variable we observed that WHR, BFM, BF%, BMI and adiponectin were significant predictors in univariate analysis. In multiple regression analysis WHR and adiponectin were independent predictors of hsCRP.

***Conclusion:*** Obese individuals have significantly higher levels of hsCRP levels and lower adiponectin levels than non obese subjects. Serum adiponectin levels and WHR are independant predictors of hsCRP levels in normoglycemic subjects.

## Introduction

 It is apparent that obesity represents one of the foremost contributory factors leading to diabetes.^1^ Metabolic syndrome is associated with high risk for the development of diabetes and cardiovascular diseases (CVD).^2^ The greater risk of type 2 Diabetes Mellitus (DM) in the obese can, at least partly, be explained by changes in adipose tissue function.^3^^-^^5^ It is now well known that adipose tissues are active endocrine tissues that produce >50 adipokines, including leptin, adiponectin, resistin, interleukin-6 (IL-6), tumor necrosis factor-α (TNF-α), interleukin-1β (IL-1β), vascular endothelial growth factors, nerve growth factors, and haptoglobin.^6^

 The recent interests in adipocyte derived factors have resulted in identification of a large group of adipocyte specific proteins in blood, such as adiponectin, acylation stimulating proteins, resistin and leptin.^7^ These adipocyte derived hormones are presently under intensive investigations concerning their involvement in the regulation of adipose tissue physiology, and in particular, their potential implication in insulin resistance, obesity and diabetes.^8^ Resistin is a protein hormone secreted by adipocytes which leads to insulin resistance and is considered to be an important link between obesity and diabetes.^9^ Resistin has been involved in the pathogenesis of obesity-mediated insulin resistance and type 2 DM. The expression and functional properties of adipocytokines and their effects on metabolism have been under intensive investigation. Indeed, studies on adipocytokines and their potential effects in human obesity and type 2 DM have implicated them in the pathogenesis of metabolic syndrome.^10^ However, still the true pathogenic mechanism that leads to accelerated atherogenesis in DM is still not known, because of its multi system ramifications. Elevated Lipoprotein(a) [Lp(a)] levels confer genetic predisposition to CVD and may be one of the links to accelerated atherogenesis in DM.^11^^-^^13^ Our previous reports have shown that Lp(a) is a marker of the presence, diffuseness and severity of CAD in Saudi adults.^14^

 A large body of evidence have shown that the inflammatory biomarker high-sensitivity C-reactive protein (hsCRP) is an independent predictor of cardiovascular events and it also predicts risk of incident hypertension and diabetes.^15^ Although it is apparent that inconsistencies remain in the data for a role of adipokines in obesity, there is a compelling evidence for their involvement in the etiology of insulin resistance and type 2 DM. The relationship among obesity, inflammatory markers (such as adipokines, phase reactant proteins), and insulin resistance (IR) has been investigated extensively in several populations but not explicitly in Saudi arabian population in which the prevalence of obesity and associated comorbidities is one of the highest in the world. Men have considerably lower plasma adiponectin levels than women, concordant with previous studies.^16^ Therefore we studied only one gender to exclude the possible confounding effect of gender on variables studied. This may partly relate to inhibitory effects of androgens on adiponectin levels.^17^

 To the best of our knowledge there are few studies relating both resistin and adiponectin in normoglycemic subjects with obesity indices. Therefore, this project evaluated the phenotypic relationship between obesity indices, resistin, adiponectin and cardiovascular risk markers in normoglycemic healthy individuals.

## Methodology

***Subjects: ***This cross-sectional study was conducted in the Department of Physiology College of Medicine, King Saud University, Riyadh. Patients were recruited from King Khalid University Hospital. The consent form was designed both in Arabic and English and was approved by the College of Medicine ethics review board. A total of 120 male subjects were selected for the study. All subjects underwent Body composition analysis.

***Laboratory Analysis: ***Overnight fasting blood samples were collected, and analyzed for fasting blood glucose (FBG), total cholesterol (TC), Triglycerides (TG), Low density Lipoprotein (LDL), High density lipoprotein (HDL), adiponectin, resistin, Lp(a) and hsCRP. Human hsCRP and Lp(a) immunoassays were performed by quantitative standard sandwich ELISA technique using monoclonal antibody specific for these analytes with kits supplied by IBL International GMBH Germany. The results of patients with hsCRP values >10 mg/L were discarded and were re-evaluated after 2-3 weeks. We followed the guidelines of the American Heart Association for measurement, evaluation and expression of hsCRP.^18^

***Statistical Analysis: ***The data was analyzed by computer software program Statistical Package for Social Sciences (SPSS Version 19, Chicago, IL). Descriptive characteristics of the study patients were calculated as mean±SD (Standard Deviation) for continuous variables. The tests applied for statistical analysis were Student’s t test for normally distributed and non-parametric Mann-Whitney U test for data which was not following normal distribution. Univariate correlations of Serum hsCRP with body indices, fat mass and other metabolic parameters were evaluated with both Spearman’s rank order and Pearson correlations. Stepwise multiple regression analyses were performed to further indentify the most significant variables contributing to hsCRP levels. A p value of ≤0.05 was taken as statistically significant.

## Results

 Anthropometric and biochemical profiles of participants included in this study are displayed in Table-I with correlation coefficients with hsCRP. We observed that BMI (r=0.326, p<0.001), BFM (r=0.377, p<0.001), BF% (r=0.326, p<0.001), WHR (r=0.402, p<0.001), Insulin (r=0.217, p=0.018) were positively correlated with hsCRP. However, serum adiponectin levels (r=0.189, p=0.0391) were negatively correlated with hsCRP.

**Table-I:** Anthropometric and metabolic characteristics of the subjects and correlation coefficients with hsCRP.

*Total Subjects N=120*	*Mean±SD*	*Minimum*	*Maximum*	*r*	*p value*
Age years	40.16±11.81	21.00	71.00	0.186	0.044
Height cm	167.42±8.21	147.00	187.00	-0.174	0.059
Weight kg	78.85±14.17	45.80	125.00	0.160	0.084
BMI kg/m2	28.13±4.80	15.60	42.30	0.326	<0.001
Fat Mass kg	24.08±8.58	6.20	52.70	0.351	<0.001
% FAT	30.01±7.58	12.50	49.80	0.377	<0.001
WHR	0.95±0.07	0.77	1.16	0.402	<0.001
FBS mmol/l	5.04±0.91	2.90	5.60	-0.047	0.617
Insulin uIU/ml	24.20±11.87	14.15	132.15	0.217	0.018
HOMA-IR	5.50±3.35	2.58	32.30	0.130	0.159
HbA1c %	5.01±0.60	2.90	5.91	-0.021	0.833
TG mmol/l	1.14±0.72	1.29	6.20	0.115	0.281
TC mmol/l	4.64±0.91	2.90	6.20	0.119	0.267
HDL mmol/l	1.19±0.22	0.75	1.63	-0.073	0.504
LDL mmol/l	2.83±0.79	1.61	4.48	0.029	0.786
Adiponectin ng/ml	127.42±58.28	36.72	308.25	-0.115	0.216
Resistin ng/ml	2.40±1.14	0.57	5.58	0.088	0.342
Lp(a) mg/dl	24.45±20.55	2.00	90.04	0.106	0.252
hsCRP mg/L	3.75±2.36	0.81	9.80		

Data are expressed as Mean±SD with ranges.

BMI, body mass index; % Fat, percent fat mass; HOMA-IR, homeostasis model assessment of insulin resistance; FBG, fasting blood glucose; TC, total cholesterol; TG, Triglycerides; LDL, Low density Lipoprotein; HDL, High density lipoprotein; Lp(a), Lipoprotein(a); high-sensitivity C-reactive protein (hsCRP)

Table-II shows comparison of Anthropometric and biochemical profiles, adipokines and CV risk markers between obese and non obese subjects. It was observed that obese subjects had siognificantly higher FBS (p=0.0466), HOMA-IR (p=0.0002), HbA1c (p=0.0366), TG (p=0.0198), hsCRP (p=0.0096) levels. While adiponectin levels were significantly lower in obese subjects compared to non obese subjects (p=0.0551). 

**Fig.1:**
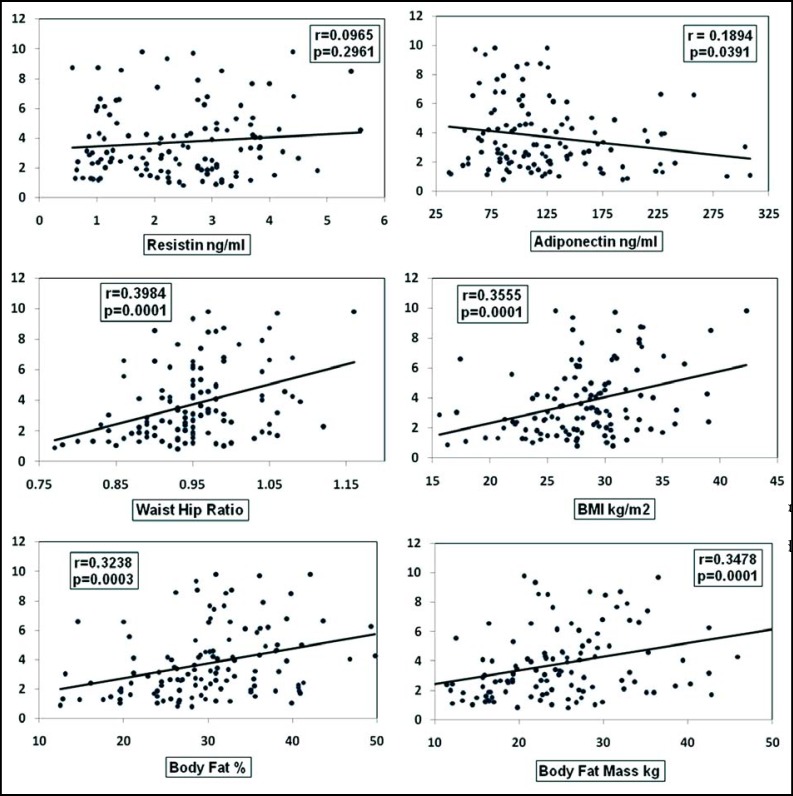
Linear regression analysis of hsCRP (mg/L) as dependant variable (Y axis) with body indices and adipokines as independent variables (X axis).

 In univariate regression analysis keeping hsCRP as dependant variable we observed that WHR, BFM, BF%, BMI and adiponectin were significant predictors However, no correlation was observed with resistin. (Fig.1) 

 The degree to which obesity markers independently predicted hsCRP was examined in a series of multiple regression models. In these models, hsCRP was the dependent variable and obesity markers, adipokines, HOMAIR and lipids were the independent variables. In multiple regression analysis many variables became non significant predictors. Only WHR and adiponectin levels turned out to be independent predictors of hsCRP and this relationship was independent of age. (Table-III)

## Discussion

 We evaluated the relationship between obesity indices (BMI, WHR, BF%, BFM), hsCRP, adipokines and IR in Saudi population. To the best of our knowledge, this study is the first to compare the associations among obesity, inflammation, adipokines and IR in Saudis. Results from this study confirmed previously reported associations between markers of obesity (BMI and WHR) and IR measured by HOMA.^19^^-^^21^

**Table-II:** Comparison of anthropometric and metabolic characteristics of between obese and non obese subjects.

	*Non Obese*	*Obese*	*P value*
	*Mean ±SD*		
Age	40.18±10.99	40.12±13.34	0.9788
Height	167±7.56	166.40±9.28	0.3224
Weight	72.53±11.18	90.28±11.68	0.0000
BMI kg/m2	25.56±3.40	32.85±2.99	0.0000
Fat Mass kg	19.63±5.71	32.11±6.89	0.0000
% FAT	26.82±6.39	35.69±6.06	0.0000
WHR	0.92±0.05	1.01±0.06	0.0000
FBS mmol/l	4.92±0.57	5.27±1.30	0.0446
Insulin uIU/ml	23.28±13.82	25.91±6.78	0.2485
HOMA-IR	4.75±1.40	5.97±1.99	0.0002
HbA1c	4.94±0.57	5.20±0.59	0.0366
TG mmol/l	1.06±0.51	1.37±0.73	0.0198
TC mmol/l	4.64±0.93	4.64±0.91	0.9757
HDL mmol/l	1.06±0.51	1.30±0.23	0.6933
LDL mmol/l	2.83±0.80	2.81±0.80	0.9017
Adiponectin ng/ml	130.04±54.91	112.53±42.84	0.0451
Resistin ng/ml	2.42±1.15	2.40±1.13	0.9351
Lp(a) mg/dl	24.79±21.95	23.85±17.98	0.8133
hsCRP mg/L	3.33±2.00	4.48±2.74	0.0096

 This study reports that serum adiponectin concentrations were inversely related to circulating hsCRP. Our data also confirms that BF% and BFM obesity are major determinants of plasma adiponectin concentrations. Higher levels of BF%, BFM and WHR, reflecting total and visceral adiposity, were associated with an additive fall in plasma adiponectin concentrations. Similar to our reports Matsushita et al.^22^ performed a cross-sectional study of 624 Japanese middle-aged men and found that adiponectin level was a more significant predictor compared to TNFa, IL-6 or hsCRP for prevalent metabolic syndrome. In line with our results previous studies have also shown that obesity, especially visceral obesity, is associated with low-grade inflammation, which is characterized by an increase in plasma concentration of inflammatory markers such as hsCRP, TNF-α and IL-6.^23^

 Consistent with previous findings, we observed significant association among hsCRP, BMI, WHR, BF% & BFM in Saudis.^24^^-^^26^ Unlike most other adipocytederived hormones, circulating adiponectin levels are decreased in obesity.^27^^,^^28^ Adiponectin increases fatty acid oxidation and directly sensitizes the body to insulin.^29^^,^^30^

 The degree to which hsCRP may be predicted by markers of obesity were also assessed in different univariate and multivariate regression models. Taken together, our model showed that adiponectin and WHR were independent predictors of hsCRP for CV risk. Following the above observation, it is reasonable to suggest that the relationship between adipokines and hsCRP is to a significant extent dependent on visceral adiposity especially WHR.

 Potential limitation of our study is its cross-sectional design from which we are not able to provide a strong causative conclusion. Secondly, our subjects were relatively homogenous with lower CVD risks because all were normoglycemic. Although various confounding factors such as sex, age and cigarette smoking have been minimized, we should be cautious to apply the conclusion to general population. Therefore, further extending the study samples to women and other ethnic populations with various CVD risks is needed.

**Table-III:** Multiple regression analysis for prediction of hsCRP in 120 healthy middle aged men.

	*Standardized Coefficients Beta*	*P value*	*95.0% Confidence Interval for B (Lower-Upper)*
Age years	-.019	.897	-.064-.057
Fat Mass kg	.575	.252	-.132-.492
Weight kg	-.688	.329	-.370-.126
Height cm	.254	.580	-.196-.347
BF%	-.218	.579	-.340-.192
WHR	.669	.011	5.474-41.305
BMI kg/m2	-.057	.913	-.627-.562
FBS mmol/dl	.410	.370	-1.941-5.138
Insulin uIU/ml	1.110	.123	-.127-1.036
HOMA-IR	-1.110	.161	-4.529-.772
HbA1c%	.026	.930	-2.202-2.406
TG mmol/L	-.038	.799	-1.087-.841
TC mmol/L	.236	.362	-.761-2.054
HDL mmol/L	-.039	.748	-3.154-2.278
LDL mmol/L	-.444	.070	-2.952-.117
Adiponectin ng/ml	-.251	.049	-.021- -.001
Resistin ng/ml	.067	.612	-.409-.688
Lp(a) mg/dl	.150	.211	-.010-.043

## Conclusions

 Obese individuals have significantly higher levels of hsCRP levels and lower adiponectin levels than non obese subjects. Serum adiponectin levels and WHR are independent predictors of hsCRP levels in normoglycemic subjects.
